# Association of physical frailty, functional dependence, dysphagia, and depression with mild cognitive impairment in hospitalized older adults

**DOI:** 10.3389/fmed.2026.1874292

**Published:** 2026-08-06

**Authors:** Wei Chen, Xuan Xiang, Lei Zhang, Kabinuer Keyimu, Xiaohui Zhou

**Affiliations:** Department of Geriatrics, The First Affiliated Hospital of Xinjiang Medical University, Urumqi, Xinjiang, China

**Keywords:** comprehensive geriatric assessment, cross-sectional study, depression, dysphagia, frailty, functional dependence, mild cognitive impairment

## Abstract

**Background:**

Geriatric syndrome assessment is of significant importance for health evaluation in hospitalized older adults. Physical frailty, functional dependence, dysphagia, and depression may coexist with mild cognitive impairment (MCI) and interact with one another.

**Methods:**

A total of 1,665 hospitalized patients aged ≥60 years from June 2024 to March 2025 were included. MCI was diagnosed according to Petersen criteria. Geriatric syndromes were assessed using the Fried Frailty Phenotype, Barthel Index (ADL), GDS-15, and the Kubota Water Swallowing Test. LASSO regression was used for variable selection, and multivariable logistic regression models were constructed. ROC curves were used to evaluate predictive performance.

**Results:**

Pre-frailty (OR = 5.90), mild ADL dependence (OR = 1.60), moderate-to-severe ADL dependence (OR = 6.01), depression (OR = 3.70), and abnormal Kubota Water Swallowing Test results (Grade II: OR = 2.71; Grade III: OR = 10.42; Grade IV/V: OR = 4.02) were all significantly associated with MCI (all *P* < 0.05). The combined model achieved an AUC of 0.773, significantly outperforming any single indicator (*P* < 0.001).

**Conclusions:**

Pre-frailty, functional dependence, depression, and dysphagia are independently and significantly associated with MCI in hospitalized older adults, with additive effects. These geriatric syndromes should serve as clinical warning signs for MCI screening, and comprehensive geriatric assessment facilitates early identification and intervention.

## 1 Introduction

With the accelerating global population aging, mild cognitive impairment (MCI), as a transitional state between normal aging and dementia, has become a major public health challenge affecting healthy aging (1). MCI not only significantly increases the risk of dementia conversion (annual conversion rate: approximately 10–15%) (2) but is also closely associated with decreased quality of life, increased caregiver burden, and healthcare resource utilization (3).

Meta-analyses have found that the prevalence of MCI in hospital-based samples is as high as 34.0% (4). However, due to the masking effects of acute illness and diffuse clinical attention, MCI is often missed or delayed in diagnosis (5). Compared with community-dwelling older adults, hospitalized older adults present with the characteristic features of “multimorbidity, polypharmacy, and functional vulnerability,” and cognitive impairment often does not occur in isolation but coexists with multiple geriatric syndromes, forming a complex clinical network (6). Acute illness not only directly threatens organ function but may also accelerate the concerted decline of multiple systems through systemic inflammation, metabolic disturbances, and immobilization-related deconditioning (7).

Comprehensive Geriatric Assessment (CGA), through multidimensional, interdisciplinary systematic evaluation, can effectively identify geriatric syndromes such as frailty, functional dependence, dysphagia, and depression, and has been established as the cornerstone of management for hospitalized older adults (8). However, systematic research on the independent associations and strength of these geriatric syndromes with MCI remains relatively limited (9). Recent studies have suggested that frailty and cognitive impairment may share a common pathophysiological foundation—“inflammaging” and neuroinflammatory pathways (10). In addition, swallowing function, as a complex behavior dependent on prefrontal cortex and subcortical executive function networks (11), raises the question of whether its abnormalities can serve as a “sentinel signal” for early cognitive impairment.

Previous studies on the association between geriatric syndromes and MCI have largely been based on community populations, with less attention paid to the highly heterogeneous group of hospitalized older adults. The hospitalized state itself constitutes a “stress challenge,” and factors such as acute illness, surgical trauma, and infection may alter the balance between geriatric syndromes and cognitive function, producing association patterns different from those in community populations (7). The bidirectional “frailty → cognitive impairment” association observed in community studies (12) may evolve into a complex “physical-cognitive” bidirectional interaction network in the hospital setting (13)—acute illness can directly impair cognitive function or indirectly affect cognition by exacerbating frailty, dysphagia, and functional dependence, while cognitive decline itself may reduce patients' ability to cope with illness, forming a vicious cycle (7, 13–15).

Therefore, this study, based on data from hospitalized older adults, aimed to: (1) analyze the strength of associations between physical frailty, functional dependence, dysphagia, and depression with MCI after controlling for demographic characteristics and biochemical indicators; (2) explore the combined predictive value of different assessment indicators for MCI; and (3) provide evidence-based support for early clinical identification of high-risk populations and the development of comprehensive intervention strategies.

## 2 Materials and methods

### 2.1 Study participants

A cross-sectional study was conducted using convenience sampling. Hospitalized older patients aged ≥60 years admitted to the Department of Geriatrics at the First Affiliated Hospital of Xinjiang Medical University from June 2024 to March 2025 were enrolled. A total of 1,665 patients were included and divided into a cognitively normal group (*n* = 1,255) and an MCI group (*n* = 410) according to MCI diagnostic criteria. This study was approved by the Ethics Committee of the First Affiliated Hospital of Xinjiang Medical University (Approval No. 240528-01). All participants or their legal representatives provided written informed consent. This study complied with the Declaration of Helsinki.

Inclusion criteria: (1) age ≥60 years; (2) generally stable condition allowing completion of assessment tests; (3) ability to cooperate with the investigation.

Exclusion criteria: (1) acute neurological disorders: delirium, acute ischemic or hemorrhagic stroke within 3 months, active central nervous system infection (meningitis, encephalitis), traumatic brain injury within 6 months; (2) progressive neurodegenerative dementias: Lewy body dementia, frontotemporal dementia, Parkinson's disease dementia, Huntington's disease; (3) structural brain lesions: brain tumors, normal pressure hydrocephalus, subdural hematoma; (4) severe systemic diseases: end-stage renal or hepatic failure, advanced cancer with brain metastases; (5) drug-induced cognitive impairment.

### 2.2 Methods

#### 2.2.1 Data collection

General information, scale assessments, inpatient diagnoses, and biochemical test results of all participants were recorded through medical record review and medical history inquiry.

#### 2.2.2 Comprehensive geriatric assessment

**Quality control**: All CGA assessments were conducted face-to-face by trained geriatricians, postgraduate students, and nurses using standardized diagnostic assessment scales, completed within 48 h of admission.

(1) General information questionnaire: Collected sociodemographic characteristics (age, sex, education level, marital status, living situation) and lifestyle factors (smoking, alcohol consumption).

(2) Neuropsychological testing: The Montreal Cognitive Assessment (MoCA) (16, 17) was used to assess executive function, verbal fluency, orientation, calculation, abstract thinking, delayed recall, visuospatial perception, naming, attention, and concentration, with a total score ranging from 0 to 30—lower scores indicating poorer cognitive function. MoCA cut-off values for MCI screening varied by education level: ≤ 13 for illiterate individuals, ≤ 19 for primary school education, and ≤ 24 for middle school education or above.

(3) Fried Frailty Phenotype: The Fried Frailty Phenotype (18) was used, comprising five components (unintentional weight loss, slow walking speed, reduced grip strength, low physical activity, and exhaustion). ≥3 components indicated frailty; 1–2 components indicated pre-frailty; 0 components indicated non-frailty.

(4) Activities of Daily Living (ADL) scale: The Barthel Index (19) was used to assess 10 items including feeding, dressing, and bathing, with a total score ranging from 0 to 100. A score of 100 indicated independence; 60–99 indicated mild dependence; 40–59 indicated moderate dependence; and < 40 indicated severe dependence.

(5) Geriatric Depression Scale (GDS-15): The GDS-15 (20) consists of 15 items, with responses of “yes” or “no.” Each “yes” response scored 1 point, and “no” scored 0, with a total score ranging from 0 to 15. Higher scores indicated more severe depressive symptoms, with ≥8 indicating depression.

(6) Kubota Water Swallowing Test result (21): Patients were seated and instructed to swallow 30 mL of warm water in one go. The swallowing time, number of swallows, and presence of coughing were observed. Grading criteria: Grade I: successful single swallow; Grade II: two or more swallows without coughing; Grade III: single swallow with coughing; Grade IV: two or more swallows with coughing; Grade V: frequent coughing, unable to swallow all water. Dysphagia was defined as Grade ≥ II. Aspiration risk was defined as Grade ≥III.

(7) Nutritional status: The NRS 2002 (22) was used, with a score ≥3 indicating nutritional risk.

(8) Pain assessment: The Numerical Rating Scale (NRS) (23) was used, with scores ranging from 0 to 10 (0 = no pain, 1–3 = mild, 4–6 = moderate, 7–10 = severe).

#### 2.2.3 MCI diagnostic criteria

According to Petersen criteria (24), the following were required:

(1) Subjective cognitive complaint;

(2) Objective cognitive impairment on MoCA;

(3) Preservation of instrumental activities of daily living (IADL), i.e., patients retained basic community living skills; this study included patients with mild-to-moderate impairment in basic ADL due to physical illness to reflect the actual functional status of hospitalized older adults;

(4) Did not meet DSM-5 criteria for dementia. All participants with MoCA scores below the cut-off were reviewed by two geriatricians.

### 2.3 Model development and construction

(1) Candidate variables were pre-screened based on clinical knowledge and literature review, with variables showing *P* < 0.05 in univariate analysis retained for further selection.

(2) LASSO (Least absolute shrinkage and selection operator) regression with 10-fold cross-validation was used to select the final predictor set. The lambda.1se value (the largest λ within one standard error of the minimum cross-validation error) was used for variable selection. Variables with non-zero coefficients at lambda.1se were retained for the final logistic regression model.

(3) Stepwise-adjusted logistic regression models were constructed.

### 2.4 Statistical analysis

Multiple imputation by chained equations (MICE) was used to handle missing biochemical data. Missing rates were: BMI (1.02%), creatinine (2.1%), triglycerides (3.36%), total cholesterol (3.3%), HDL (3.36%), LDL (3.66%), albumin (2.04%), and HbA1c (14.59%). The imputation model included all analysis variables (including the outcome variable) as predictors. Predictive mean matching (PMM) was used for continuous variables. Categorical variables were converted to factors before imputation and were included as predictors but not imputed. Five imputed datasets (m = 5) were generated with 20 iterations. Convergence was verified using trace plots, density plots, and strip plots, showing good convergence and imputed values consistent with observed distributions.

Non-normally distributed continuous variables were presented as medians (interquartile range) [M (P25, P75)], with group comparisons using the Mann-Whitney *U* test. Categorical variables were presented as frequencies and percentages (*n*, %), with group comparisons using the chi-square test. Binary logistic regression was used to analyze the associations of frailty, ADL, depression, and the Kubota water swallowing test with MCI. Model 1 was unadjusted; Model 2 adjusted for demographic characteristics; Model 3 further adjusted for biochemical variables that showed significant differences. ROC curves were used to evaluate the predictive performance of frailty, ADL, depression, and the Kubota water swallowing test for MCI. The Delong test was used to compare differences between ROC curves. *P* < 0.05 was considered statistically significant. All statistical analyses were performed using R software (version 4.6.0) [R Foundation for Statistical Computing, Vienna, Austria (https://www.R-project.org/)].

## 3 Results

### 3.1 Baseline characteristics and univariate analysis

A total of 1,665 hospitalized older patients were included, of whom 410 (24.62%) had MCI and 1,255 (75.38%) were cognitively normal. Univariate analysis showed statistically significant differences between the two groups in age, BMI, triglycerides, albumin, HbA1c, frailty, nutritional risk, ADL, depression, Kubota water swallowing test results, sex, education level, marital status, pre-retirement occupation, smoking, alcohol consumption, cerebrovascular disease, gastric disease, and Parkinson's disease (*P* < 0.05). No statistically significant differences were found in creatinine, total cholesterol, HDL, LDL, pain, living situation, hypertension, diabetes, heart disease, chronic lung disease, asthma, liver disease, kidney disease, arthritis/rheumatism, or cancer/malignancy (*P* > 0.05). Detailed results are presented in Table 1.

**Table 1:** Comparison of characteristics between cognitively normal and MCI groups.

Variables	Total (*n* = 1665)	Cognitively normal (*n* = 1255)	MCI group (*n* = 410)	Statistic	*P*
Age, *M* (Q1, Q3)	71.00 (65.00, 79.00)	70.00 (64.00, 77.00)	77.00 (70.00, 82.00)	*Z* = −10.61	**< 0.001**
BMI, *M* (Q1, Q3)	25.34 (23.00, 27.92)	25.80 (23.44, 28.41)	24.44 (22.00, 26.43)	*Z* =-7.36	**< 0.001**
Creatinine, *M* (Q1, Q3)	68.70 (57.83, 83.34)	68.59 (58.10, 81.50)	69.00 (57.10, 87.95)	*Z* =-1.13	0.258
Triglycerides, *M* (Q1, Q3)	1.21 (0.89, 1.62)	1.22 (0.90, 1.65)	1.16 (0.84, 1.55)	*Z* =-2.45	**0.014**
Total cholesterol, *M* (Q1, Q3)	3.80 (3.12, 4.56)	3.82 (3.13, 4.56)	3.73 (3.10, 4.53)	*Z* =-1.14	0.254
HDL, *M* (Q1, Q3)	1.00 (0.86, 1.17)	1.00 (0.87, 1.17)	0.99 (0.85, 1.15)	*Z* =-0.87	0.384
LDL, *M* (Q1, Q3)	2.58 (2.01, 3.21)	2.60 (2.02, 3.23)	2.50 (1.94, 3.13)	*Z* =-1.90	0.058
Albumin, *M* (Q1, Q3)	38.90 (36.50, 41.10)	39.10 (36.90, 41.20)	38.10 (35.60, 40.40)	*Z* =-5.40	**< 0.001**
HbA1c, *M* (Q1, Q3)	6.00 (5.63, 6.80)	6.00 (5.61, 6.75)	6.10 (5.70, 7.28)	*Z* =-2.39	**0.017**
Frailty, *n* (%)				χ^2^ = 220.76	**< 0.001**
Non-frailty	160 (9.61)	146 (11.63)	14 (3.41)		
Pre-frailty	581 (34.89)	314 (25.02)	267 (65.12)		
Frailty	924 (55.50)	795 (63.35)	129 (31.46)		
Nutritional risk, *n* (%)				χ^2^ = 18.90	**< 0.001**
No	1,524 (91.53)	1,170 (93.23)	354 (86.34)		
Yes	141 (8.47)	85 (6.77)	56 (13.66)		
ADL, *n* (%)				χ^2^ = 91.16	**< 0.001**
Independent	824 (49.49)	695 (55.38)	129 (31.46)		
Mild dependence	812 (48.77)	551 (43.90)	261 (63.66)		
Moderate-to-severe dependence	29 (1.74)	9 (0.72)	20 (4.88)		
Depression, *n* (%)				χ^2^ = 80.05	**< 0.001**
No	1,533 (92.07)	1,198 (95.46)	335 (81.71)		
Yes	132 (7.93)	57 (4.54)	75 (18.29)		
Pain, *n* (%)				χ^2^ = 0.53	0.466
No	460 (27.63)	341 (27.17)	119 (29.02)		
Yes	1,205 (72.37)	914 (72.83)	291 (70.98)		
Water swallowing test result, *n* (%)				χ^2^ = 140.76	**< 0.001**
Grade I	694 (41.68)	616 (49.08)	78 (19.02)		
Grade II	902 (54.17)	610 (48.61)	292 (71.22)		
Grade III	29 (1.74)	9 (0.72)	20 (4.88)		
Grade IV/Grade V	40 (2.40)	20 (1.59)	20 (4.88)		
Sex, *n* (%)				χ^2^ = 4.88	**0.027**
Male	769 (46.19)	599 (47.73)	170 (41.46)		
Female	896 (53.81)	656 (52.27)	240 (58.54)		
Education level, *n* (%)				χ^2^ = 42.71	**< 0.001**
Illiterate	100 (6.01)	55 (4.38)	45 (10.98)		
Primary school	367 (22.04)	250 (19.92)	117 (28.54)		
Middle school or above	1,198 (71.95)	950 (75.70)	248 (60.49)		
Marital status, *n* (%)				χ^2^ = 34.42	**< 0.001**
Married	1,288 (77.36)	1,014 (80.80)	274 (66.83)		
Divorced/widowed/separated/ unmarried	377 (22.64)	241 (19.20)	136 (33.17)		
Pre-retirement occupation, *n* (%)				χ^2^ = 17.92	**0.003**
Professional/technical	581 (34.89)	460 (36.65)	121 (29.51)		
Worker	325 (19.52)	234 (18.65)	91 (22.20)		
Farmer	447 (26.85)	319 (25.42)	128 (31.22)		
Civil servant	180 (10.81)	145 (11.55)	35 (8.54)		
Self-employed	37 (2.22)	32 (2.55)	5 (1.22)		
Freelancer	95 (5.71)	65 (5.18)	30 (7.32)		
Living situation, *n* (%)				χ^2^ = 1.12	0.291
Living with others (spouse/children/domestic helper/other relatives)	1,509 (90.63)	1,132 (90.20)	377 (91.95)		
Living alone	156 (9.37)	123 (9.80)	33 (8.05)		
Smoking, *n* (%)				χ^2^ = 7.40	**0.007**
No	1,526 (91.65)	1,137 (90.60)	389 (94.88)		
Yes	139 (8.35)	118 (9.40)	21 (5.12)		
Alcohol consumption, *n* (%)				χ^2^ = 11.53	**< 0.001**
No	1,535 (92.19)	1,141 (90.92)	394 (96.10)		
Yes	130 (7.81)	114 (9.08)	16 (3.90)		
Hypertension, *n* (%)				χ^2^ = 0.10	0.75
No	473 (28.41)	354 (28.21)	119 (29.02)		
Yes	1,192 (71.59)	901 (71.79)	291 (70.98)		
Diabetes, *n* (%)				χ^2^ = 3.55	0.06
No	1,119 (67.21)	859 (68.45)	260 (63.41)		
Yes	546 (32.79)	396 (31.55)	150 (36.59)		
Heart disease, *n* (%)				χ^2^ = 3.51	0.061
No	483 (29.01)	379 (30.20)	104 (25.37)		
Yes	1,182 (70.99)	876 (69.80)	306 (74.63)		
Cerebrovascular disease, *n* (%)				χ^2^ = 10.66	**0.001**
No	679 (40.78)	540 (43.03)	139 (33.90)		
Yes	986 (59.22)	715 (56.97)	271 (66.10)		
Chronic lung disease, *n* (%)				χ^2^ = 0.14	0.704
No	1,321 (79.34)	993 (79.12)	328 (80.00)		
Yes	344 (20.66)	262 (20.88)	82 (20.00)		
Asthma, *n* (%)				χ^2^ = 0.17	0.677
No	1,611 (96.76)	1,213 (96.65)	398 (97.07)		
Yes	54 (3.24)	42 (3.35)	12 (2.93)		
Gastric disease, *n* (%)				χ^2^ = 4.81	**0.028**
No	1,423 (85.47)	1,059 (84.38)	364 (88.78)		
Yes	242 (14.53)	196 (15.62)	46 (11.22)		
Liver disease, *n* (%)				χ^2^ = 0.05	0.824
No	1,607 (96.52)	1,212 (96.57)	395 (96.34)		
Yes	58 (3.48)	43 (3.43)	15 (3.66)		
Kidney disease, *n* (%)				χ^2^ = 2.68	0.102
No	1,471 (88.35)	1,118 (89.08)	353 (86.10)		
Yes	194 (11.65)	137 (10.92)	57 (13.90)		
Parkinson's disease, *n* (%)				χ^2^ = 10.75	**0.001**
No	1,643 (98.68)	1,245 (99.20)	398 (97.07)		
Yes	22 (1.32)	10 (0.80)	12 (2.93)		
Arthritis or rheumatism, n (%)				χ^2^ = 0.62	0.43
No	1,597 (95.92)	1,201 (95.70)	396 (96.59)		
Yes	68 (4.08)	54 (4.30)	14 (3.41)		
Cancer or malignancy, *n* (%)				χ^2^ = 0.81	0.369
No	1,574 (94.53)	1,190 (94.82)	384 (93.66)		
Yes	91 (5.47)	65 (5.18)	26 (6.34)		

Z: Mann-Whitney test, χ^2^: Chi-square test; M: Median, Q1: 1st Quartile, Q3: 3rd Quartile.

Bold: *P* < 0.05.

### 3.2 LASSO regression variable selection

**Figure 1:**
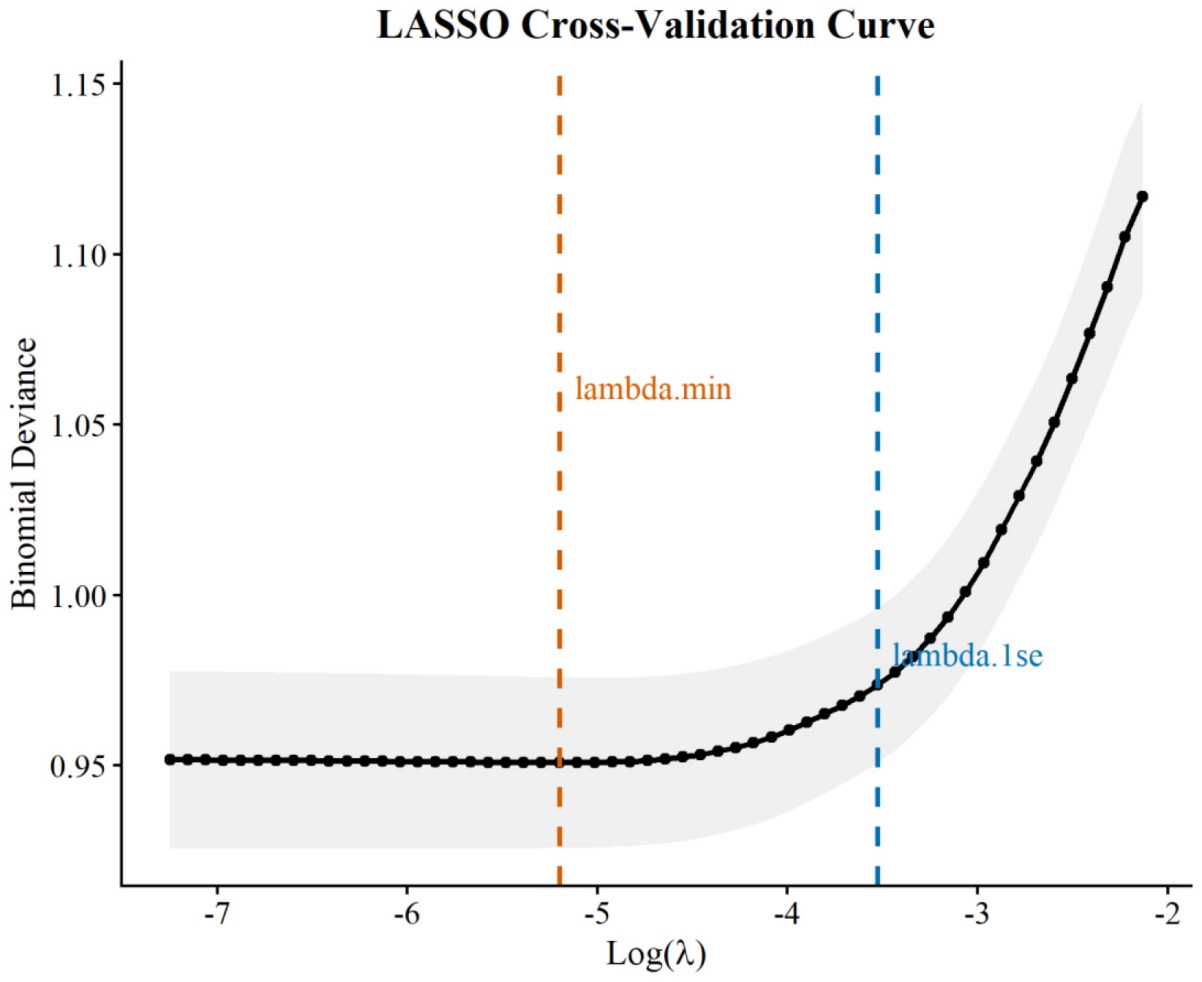
Ten-fold cross-validation plot of the Lasso regression model.

**Figure 2:**
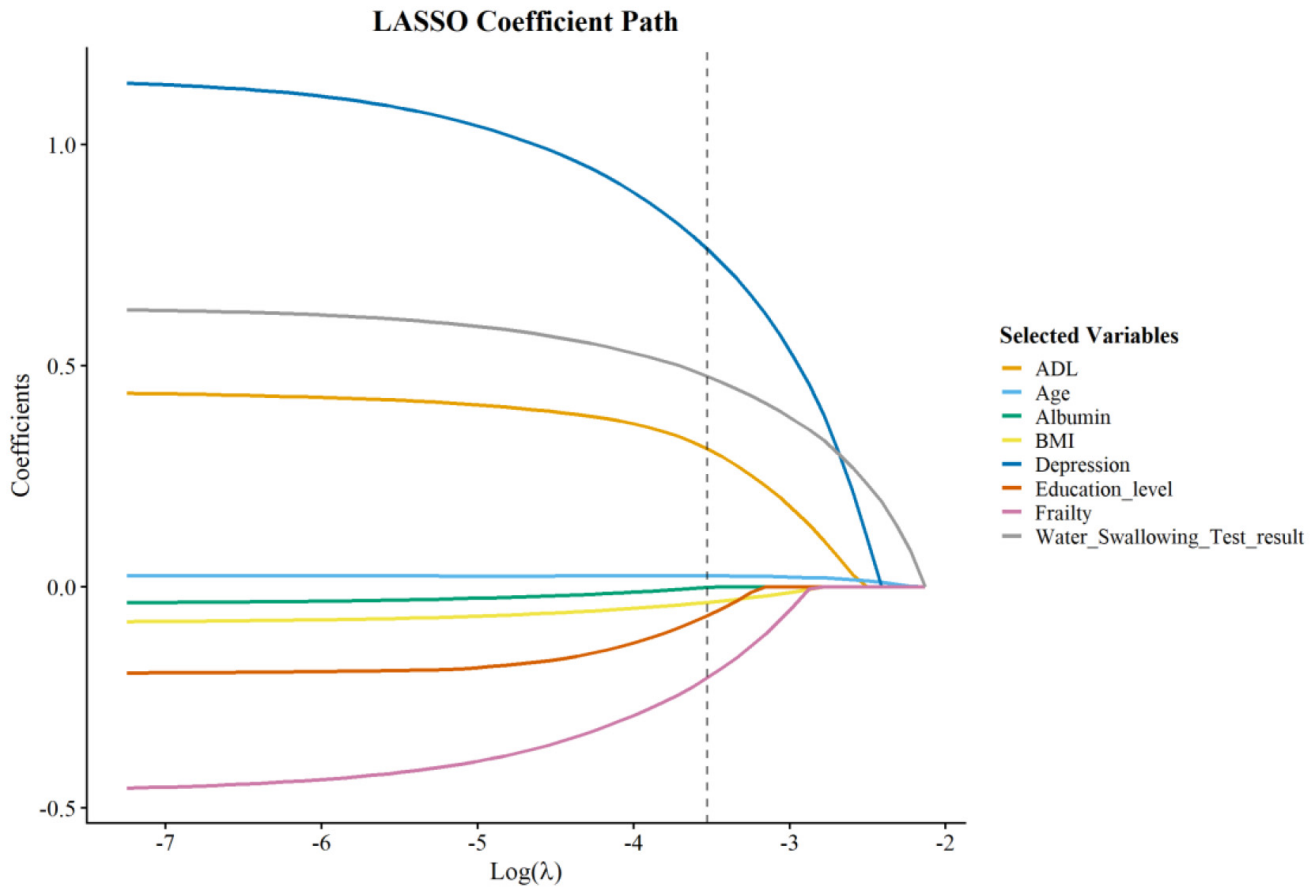
Lasso regression path plot.

Variables with statistically significant differences in univariate analysis were included in the LASSO regression model. Ten-fold cross-validation was used to select the optimal penalty parameter λ (lambda.1se). The model selected the following variables: frailty, ADL, depression, Kubota water swallowing test result, age, education level, BMI, and albumin. These variables were included in the subsequent multivariable logistic regression models. The LASSO cross-validation plot and path plot are shown in Figures 1 and 2, respectively.

### 3.3 Variable assignment and collinearity diagnostics

Using MCI as the dependent variable, the variables selected by LASSO regression were included in the multivariable logistic regression analysis. All variables had variance inflation factors (VIF) < 5 and tolerance >0.1, indicating no significant multicollinearity. Variable assignments are detailed in Table 2.

**Table 2:** Variable assignment.

Variable	Assignment
MCI	0 = No, 1 = Yes
Frailty	0 = Non-frailty, 1 = Pre-frailty, 2 = Frailty
ADL	0 = Independent, 1 = Mild dependence, 2 = Moderate-to-severe dependence
Depression	0 = No, 1=Yes
Kubota Water Swallowing Test	1 = Grade I, 2 = Grade II, 3 = Grade III, 4 = Grade IV/V
Age	Continuous variable
Education level	1 = Illiterate, 2 = Primary school, 3 = Middle school or above
BMI	Continuous variable
Albumin	Continuous variable

### 3.4 Multivariable logistic regression analysis

Three stepwise-adjusted logistic regression models were constructed with MCI as the dependent variable. Model 1 was unadjusted; Model 2 adjusted for age, education level, and BMI; Model 3 further adjusted for albumin. Results are shown in Table 3.

**Table 3:** Logistic regression analysis of geriatric syndromes affecting MCI.

Variable	Subgroup	Model 1		Model 2		Model 3	
		OR(95%CI)	*P*	OR(95%CI)	*P*	OR(95%CI)	*P*
Frailty							
	Non-frailty	1.00		1.00		1.00	
	Pre-frailty	8.87 (5.18–16.40)	**< 0.001**	6.11(3.47–11.52)	**< 0.001**	5.90 (3.35–11.15)	**< 0.001**
Frailty		1.69 (0.98–3.15)	0.075	1.46(0.84–2.73)	0.210	1.44 (0.83–2.70)	0.223
ADL							
	Independent	1.00		1.00		1.00	
	Mild dependence	2.55 (2.01–3.25)	**< 0.001**	1.66 (1.27–2.18)	**< 0.001**	1.60 (1.22–2.10)	**< 0.001**
	Moderate-to-severe dependence	11.97 (5.48–28.20)	**< 0.001**	6.38 (2.83–15.41)	**< 0.001**	6.01 (2.66–14.55)	**< 0.001**
**Depression**							
	No	1.00		1.00		1.00	
	Yes	4.71 (3.27–6.80)	**< 0.001**	3.75 (2.54–5.55)	**< 0.001**	3.70 (2.51–5.48)	**< 0.001**
Water swallowing test result							
	Grade I	1.00		1.00		1.00	
	Grade II	3.78 (2.89–5.00)	**< 0.001**	2.70 (2.03–3.62)	**< 0.001**	2.71 (2.03–3.63)	**< 0.001**
	Grade III	17.55 (7.94–41.81)	**< 0.001**	10.49 (4.63–25.48)	**< 0.001**	10.42 (4.59–25.36)	**< 0.001**
	Grade IV/Grade V	7.90 (4.06–15.40)	**< 0.001**	4.08 (2.01–8.24)	**< 0.001**	4.02 (1.98–8.13)	**< 0.001**

Model 1: unadjusted; Model 2: adjusted for age, education level, and BMI; Model 3: adjusted for age, education level, BMI, and albumin. Bold: *P* < 0.05.

#### 3.4.1 Frailty

Compared with non-frailty, pre-frailty was significantly associated with increased MCI risk (Model 3: OR = 5.90, 95% CI: 3.35–11.15, *P* < 0.001). The association between frailty and MCI was not statistically significant (Model 3: OR = 1.44, 95% CI: 0.83–2.70, *P* = 0.223).

#### 3.4.2 ADL

Compared with independence, mild dependence (Model 3: OR = 1.60, 95% CI: 1.22–2.10, *P* < 0.001) and moderate-to-severe dependence (Model 3: OR = 6.01, 95% CI: 2.66–14.55, *P* < 0.001) were both significantly associated with MCI.

#### 3.4.3 Depression

Depression was significantly associated with increased MCI risk (Model 3: OR = 3.70, 95% CI: 2.51–5.48, *P* < 0.001).

#### 3.4.4 Kubota water swallowing test

Compared with Grade I, Grades II, III, and IV/V were all significantly associated with increased MCI risk (Model 3: OR = 2.71, 10.42, and 4.02, respectively; 95% CI: 2.03–3.63, 4.59–25.36, and 1.98–8.13, respectively; all *P* < 0.001).

### 3.5 Predictive performance of the combined assessment model

**Figure 3:**
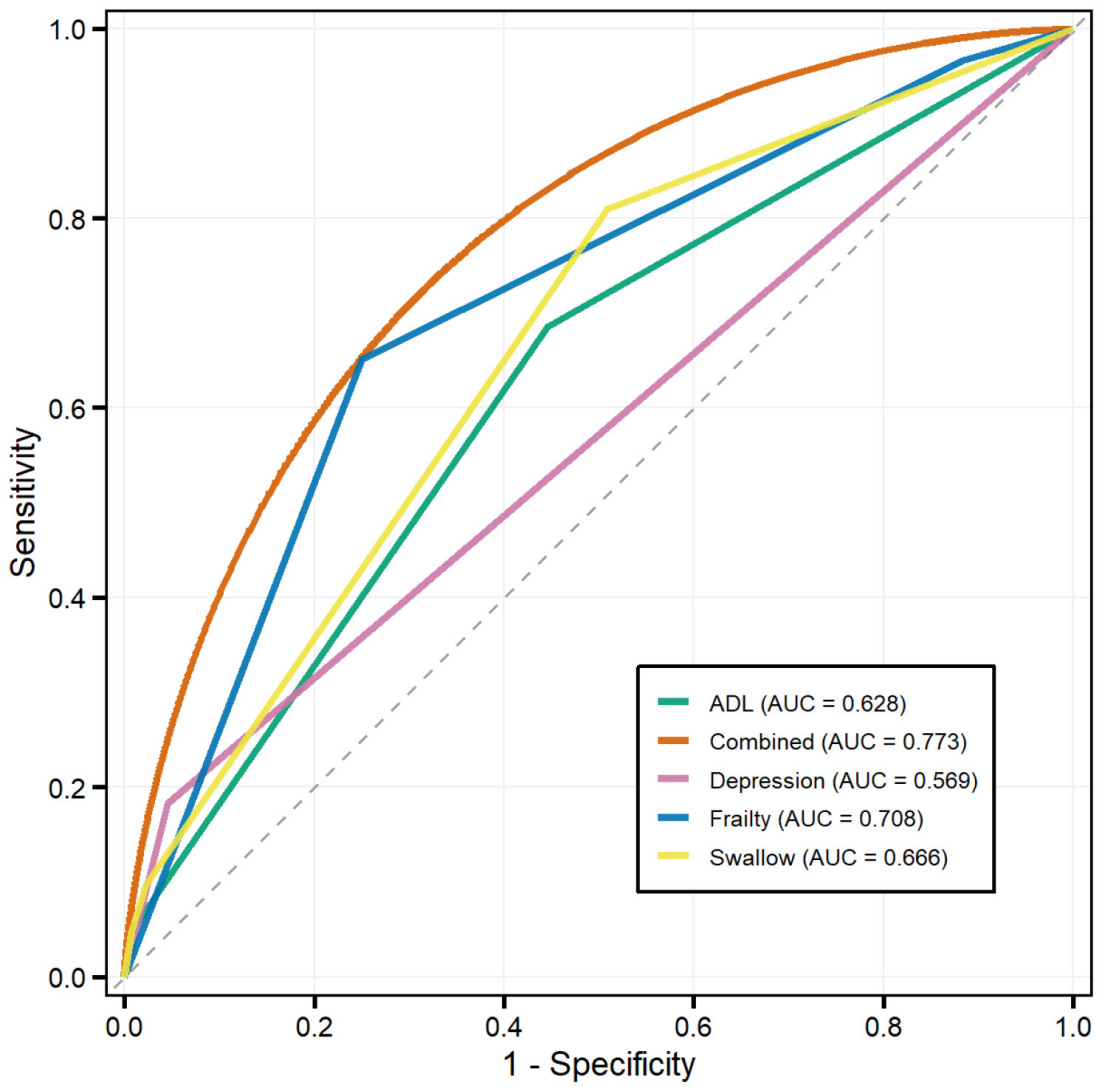
Comparison of AUC under ROC curves for individual and joint models.

ROC curves were plotted to compare the predictive performance of different assessment indicators for MCI. The combined model (including frailty, ADL, depression, and Kubota water swallowing test) achieved an AUC of 0.773, significantly higher than any single-indicator model (frailty: 0.708; ADL: 0.628; depression: 0.569; Kubota water swallowing test: 0.666). Delong tests showed that all differences were statistically significant (all *P* < 0.001). See Figure 3 and Table 4.

**Table 4:** Comparison of areas under the ROC curves.

Comparison	AUC_Model1	AUC_Model2	AUC difference	*Z*	*P*
Combined vs. frailty	0.773	0.708	0.065	8.48	< 0.001
Combined vs. ADL	0.773	0.628	0.145	10.73	< 0.001
Combined vs. Depression	0.773	0.569	0.204	14.77	< 0.001
Combined vs. Kubota Water Swallowing test	0.773	0.666	0.107	9.22	< 0.001

## 4 Discussion

This study, based on cross-sectional data from 1,665 hospitalized older adults, systematically analyzed the independent associations of physical frailty, functional dependence, dysphagia, and depression with mild cognitive impairment. After adjusting for age, education level, BMI, and albumin, pre-frailty (OR = 5.90), ADL dependence (mild: OR = 1.60; moderate-to-severe: OR = 6.01), depression (OR = 3.70), and abnormal Kubota water swallowing test results (Grade II: OR = 2.71; Grade III: OR = 10.42; Grade IV/V: OR = 4.02) were all independently and significantly associated with MCI, with clear additive effects—the combined model achieved an AUC of 0.773, significantly outperforming any single indicator. These findings are consistent with multiple international studies (25–32), suggesting that geriatric syndromes do not affect cognitive function in isolation but may have cumulative predictive effects through interconnected pathways to is associated with the progression of the development and progression of cognitive impairment.

This study found that pre-frailty had the strongest association with MCI (OR = 5.90), consistent with the trend observed by Nyunt et al. (33) in the Singapore Longitudinal Aging Study, although with a higher effect size (OR = 5.90 vs. 1.67), likely related to the acute stress state in hospitalized populations. The association between frailty and MCI was not statistically significant after adjusting for confounders (OR = 1.44, 95% CI: 0.83–2.70, *P* = 0.223). This counter-intuitive finding prompted us to re-examine the applicability of frailty assessment tools in the hospital setting.

From a pathophysiological perspective, although individuals with pre-frailty do not yet meet the Fried frailty criteria, their physiological systems are already in a state of “subclinical reserve decline” (34). The inflammaging theory posits that persistent elevation of low-grade systemic inflammatory factors such as IL-6 and TNF-α can cross the blood-brain barrier to activate microglia, impair hippocampal synaptic plasticity, and thereby accelerate cognitive decline (35). Individuals with pre-frailty may be in the early stages of this pathological process—inflammatory factors have begun to accumulate but remain within the compensatory range; hospitalization stress may break this compensatory balance, bringing cognitive impairment from a subclinical to a manifest state (36). In contrast, frail patients, due to prolonged bed rest, polypharmacy, and severe functional impairment, may have their cognitive assessments more heavily confounded by somatic symptoms (such as delirium, inattention, and poor cooperation), leading to information bias in MCI detection—some patients with genuine cognitive impairment may be excluded due to inability to complete the full assessment, while others may be misclassified due to acute encephalopathy, thus diluting the true association.

Among the five components of the Fried Frailty Phenotype, slow walking speed and low physical activity are particularly susceptible to acute illness in the hospital setting. Hospitalized older adults, even with intact cognitive function, often exhibit reduced walking speed and activity due to acute infection, surgical trauma, or immobilization, and may therefore be classified as “frail” by the Fried criteria. This “acute illness-related phenotype” may differ fundamentally from the “frailty phenotype” reflecting chronic physiological reserve depletion in community populations—the former being reversible and stress-driven, the latter irreversible and cumulative. The “paradoxical” finding that the proportion of frailty in the cognitively normal group (63.35%) was higher than in the MCI group (31.46%) supports this interpretation. From a clinical translation perspective, pre-frailty may have greater clinical utility than frailty as a “warning signal” for MCI—it suggests that intervention during the compensatory phase of cognitive function may be more effective in blocking the progression from frailty to cognitive impairment.

This study found that Grades II, III, and IV/V of the Kubota water swallowing test were associated with 2.71-, 10.42-, and 4.02-fold increased risks of MCI, respectively, demonstrating a non-linear association with a threshold effect. The sharp increase from 2.71-fold at Grade II to 10.42-fold at Grade III suggests a possible “threshold effect” in cognitive impairment as swallowing function transitions from “compensated abnormality” (Grade II: multiple swallows without coughing) to “decompensated abnormality” (Grade III and above: coughing or inability to complete). This association remained robust even after adjusting for age and albumin.

Swallowing is not an isolated motor behavior; its initiation and coordination are highly dependent on the prefrontal cortex, anterior cingulate cortex, and subcortical executive function networks (37). A complete swallowing action requires precise coordination of sensory input, cortical integration, motor planning, and execution—damage to any of these links may affect swallowing function. Therefore, swallowing abnormalities may indicate coexisting cerebral small vessel disease or early neurodegenerative changes, pathological processes that also impair cognitive function. A Japanese community-based study (38) found that frailty was independently associated with worsened swallowing function (OR = 2.56), while MCI was independently associated with reduced tongue pressure (OR = 1.77), further supporting the close physiological connection between oral motor function and cognitive function.

At the mechanistic level, the association between dysphagia and MCI may involve three pathways: (1) Shared pathological pathway—cerebral small vessel disease simultaneously damages cortical swallowing centers and cognitive-related brain regions (39); (2) Malnutrition-inflammation pathway—dysphagia is closely related to aspiration, recurrent pulmonary infection, and malnutrition, indirectly impairing cognition through systemic inflammation and metabolic disturbances (40); (3) Early neurodegenerative manifestation—delayed swallowing initiation may reflect early executive dysfunction, serving as a “motor sentinel sign” of MCI (41). This finding has direct clinical screening implications: patients with abnormal Kubota water swallowing test results, even without subjective cognitive complaints, should be included in MCI screening.

In this study, both mild ADL dependence (OR = 1.60) and moderate-to-severe ADL dependence (OR = 6.01) were significantly associated with MCI, with effect sizes increasing with the severity of dependence, demonstrating a clear gradient effect consistent with the findings of Hendlmeier et al. (42). This gradient effect suggests that even mild ADL dependence (Barthel Index 60–99) is already associated with significantly increased MCI risk, indicating that subtle changes in functional status may be early signals of cognitive impairment in hospitalized older adults.

The “preservation of basic activities of daily living” required by the Petersen MCI criteria refers to instrumental activities of daily living (IADL)—the ability to manage finances, shop, use the telephone, and other community living skills. In contrast, the Barthel Index used in this study assesses basic activities of daily living (BADL)—fundamental self-care activities such as feeding, dressing, and toileting. Among the hospitalized older adults included in this study, some had mild-to-moderate Barthel Index dependence due to acute illness or chronic comorbidities, while their IADL may not yet have reached the level of dementia-related impairment. Therefore, the ADL assessment (Barthel Index) in this study does not constitute logical overlap or circular reasoning with the MCI diagnostic criteria (IADL preservation). Rather, this study reveals that even when cognitive function is only at the MCI stage, decline in basic ADL has already become a clinically notable signal in the context of physical illness.

Depression was also associated with a 3.70-fold increased risk of MCI, consistent with the findings of Ding et al. (43) based on ADNI data, which showed that persistent high depressive symptoms increased the risk of MCI-to-AD conversion by 3.79-fold (HR = 3.79). Depression is an independent risk factor for MCI, and more severe depressive symptoms are associated with higher risks of cognitive impairment or dementia conversion.

Although the cross-sectional design precludes determination of causal direction, these variables may exhibit mutually reinforcing association patterns with MCI: on one hand, frailty, low albumin, and low BMI lead to muscle loss and systemic inflammation, which are associated with cognitive impairment and subsequently with ADL dependence and depression; on the other hand, early cognitive decline may be associated with IADL impairment and reduced emotional regulation, manifesting as mild functional dependence and depressive symptoms. Cognitive impairment and functional decline may form a “self-reinforcing cycle”—cognitive decline limits functional independence, and functional limitation reduces cognitive stimulation and social participation, accelerating cognitive deterioration. Depression serves as both a catalyst (exacerbating cognitive and functional impairment) and a product in this cycle, acting as a “bidirectional amplifier.”

This association pattern has direct clinical implications: even without meeting MCI diagnostic criteria, newly emerging ADL dependence or depressive symptoms in hospitalized older adults should be considered “warning signals” of cognitive deterioration. Conversely, for patients already diagnosed with MCI, routine assessment of ADL and emotional status should be performed, with targeted rehabilitation and psychological interventions provided. Management of depression may have a “leveraging effect”—improving emotional state may break the “cognition-function” vicious cycle, creating favorable conditions for cognitive rehabilitation.

This study found that both BMI and albumin were negatively associated with MCI, consistent with the findings of Ikeuchi et al. (44), and these associations were independent of frailty and swallowing function, suggesting that the malnutrition-inflammation state may be the “common soil” connecting physical frailty and cognitive impairment. This finding is consistent with the “muscle-brain axis” hypothesis (45): muscle tissue, as an endocrine organ, secretes “brain-beneficial factors” such as irisin and brain-derived neurotrophic factor (BDNF) during contraction. Hospitalized older adults experience increased catabolism and accelerated muscle loss due to acute illness, reducing the secretion of protective factors while promoting the release of pro-inflammatory cytokines such as IL-6 and TNF-α, impairing hippocampal-dependent memory function (46, 47). Low albumin reflects systemic inflammation or malnutrition, further supporting the “nutrition-inflammation-cognition axis” (48).

Age is the most important non-modifiable risk factor for MCI (49–52). Normal aging is associated with approximately 0.5% annual brain volume loss (particularly in the hippocampus and prefrontal cortex), and the plasticity of brain networks declines with age (53). The significantly higher median age in the MCI group (77 years) compared with the cognitively normal group (70 years) in this study confirms the significance of age effects. Education level is an independent protective factor for MCI. The cognitive reserve hypothesis (54) suggests that individuals with higher education have more developed neuronal networks and greater compensatory capacity, enabling more effective resistance to the clinical expression of neuropathological damage. In the hospital setting, education level may also indirectly protect cognitive function by influencing health literacy, treatment adherence, and medical decision-making capacity. This finding suggests that assessment of cognitive reserve should be an integral component of cognitive screening in hospitalized older adults, with those with lower education potentially requiring lower cognitive assessment cutoffs or more closely monitored cognitive follow-up.

## 5 Limitations

This study has several limitations. First, the cross-sectional design precludes determination of causal relationships; longitudinal studies are necessary to establish causal directions. Second, the single-center design limits generalizability; external validation across different regions and hospital levels is crucial for generalizing the findings. Third, the lack of systematic recording of anticholinergic medication use, sedative use, and severity of acute infection (e.g., CRP, PCT) may have resulted in residual confounding. Fourth, the study did not distinguish between amnestic and non-amnestic MCI, nor did it include neuroimaging or genetic markers, limiting the etiological specificity of the identified associations. Fifth, the Fried Frailty Phenotype may be confounded by acute illness in hospitalized patients; future studies should consider frailty assessment tools better suited to the hospital setting (e.g., Clinical Frailty Scale, FRAIL scale) or combine them with objective inflammatory markers. Sixth, the sample size for the Grade IV/V combined group in the Kubota water swallowing test remained relatively limited (*n* = 40), and the effect estimates require validation in larger samples.

## 6 Conclusions

The detection rate of MCI in hospitalized older adults was relatively high (24.62%). This study found that pre-frailty, ADL dependence (mild and moderate-to-severe), depression, and Grade II or higher on the Kubota water swallowing test were independently and significantly associated with MCI, and combined assessment demonstrated good predictive performance for MCI (AUC = 0.773). These geriatric syndromes may have complex multidimensional associations with MCI, and their specific directions and mechanisms require further elucidation through prospective cohort studies. In clinical practice, these geriatric syndromes should be considered “warning signals” for MCI risk in hospitalized older adults, and comprehensive assessment and intervention should be implemented—not only targeting cognition itself but also focusing on breaking the vicious cycle of “cognition-function-nutrition-emotion.”

## Data Availability

The original contributions presented in the study are included in the article/supplementary material, further inquiries can be directed to the corresponding author.
